# Survival of community-acquired Bacillus cereus sepsis with venous sinus thrombosis in an immunocompetent adult man – a case report and literature review

**DOI:** 10.1186/s12879-023-08176-1

**Published:** 2023-04-06

**Authors:** Zengrong Wang, Han Xia, Fangfang Fan, Jin Zhang, Hong Liu, Jing Cao

**Affiliations:** 1 Department of Critical Care Medicine, The First Hospital of Shanxi Medical University, Taiyuan, 030001 China; 2 Department of Scientific Affairs, Hugo Biotech Co., Ltd., No.1, East Disheng Road, Beijing, 100176 China; 3 Department of Respiratory and Critical Care Medicine, The First Hospital of Shanxi Medical University, Taiyuan, 030001 China; 4 Department of Neurology, The First Hospital of Shanxi Medical University, Taiyuan, 030001 China

**Keywords:** *Bacillus cereus*, Sepsis, Venous sinus thrombosis

## Abstract

**Background:**

*Bacillus cereus* infections in immunocompetent patients are uncommon and mainly observed in fragile patients. It can cause lethal infections with multiple organ dysfunction syndrome (MODS). However, a patient presenting as venous sinus thrombosis and survival without sequela has not been reported.

**Case presentation:**

A 20-year-old previously healthy male developed gastroenteritis after a meal, followed by fever, convulsions, and severe disturbance of consciousness. The patient had significant leukocytosis with a mildly elevated D-dimer, creatinine level, and respiratory failure. The CT(computed tomography) revealed fatal brain edema and subarachnoid hemorrhage. Previous blood culture in a local hospital revealed *B. cereus*, which was confirmed by mNGS(metagenomic next-generation sequencing) using blood and urine in our hospital. Accordingly, *B. cereus* sepsis with MODS were considered. Later, cerebral venous sinus thrombosis was proved. After anti-infection (linezolid 0.6 g, Q12h; and meropenem 1.0 g, Q8h), anti-coagulant (enoxaparin 6000U, Q12h), and other symptomatic treatments, the patient recovered completely without sequela at the 6-month follow-up.

**Conclusions:**

This case suggests that in immunocompetent adults, there is still a risk of infection with *B. cereus*, causing severe MODS. Special attention should be paid to venous sinus thrombosis and subarachnoid hemorrhage in such cases, while, anti-coagulant is essential therapy.

## Background

*Bacillus cereus* (*B. cereus*) is a common gram-positive, aerobic and facultative anaerobic bacillus widely distributed in soil, water, air and intestine [1]. *B. cereus* is usually non-pathogenic and considered as a contaminating bacteria in clinical practices, but it can also cause food poisoning with self-limiting symptoms, such as vomiting and diarrhea. In immunosuppressed people, severe infectious symptoms by *B. cereus* might occur [2]. Several studies have reported that the bacterium can cause severe bacteremia, leading to multiple organ failure, especially in patients with hematological malignancies. The delay in treatment may result in an even poor prognosis [3, 4]. Here, we report a previously healthy young patient with *B. cereus* sepsis, complicated by central nervous system involvement, multiple organ dysfunction, and venous sinus thrombosis, in addition, a patient presenting as venous sinus thrombosis and survival without sequela has not been reported. A relevant literature review on *B. cereus* infection in previously healthy individuals has also been provided.

## Case presentation

**Fig. 1:**
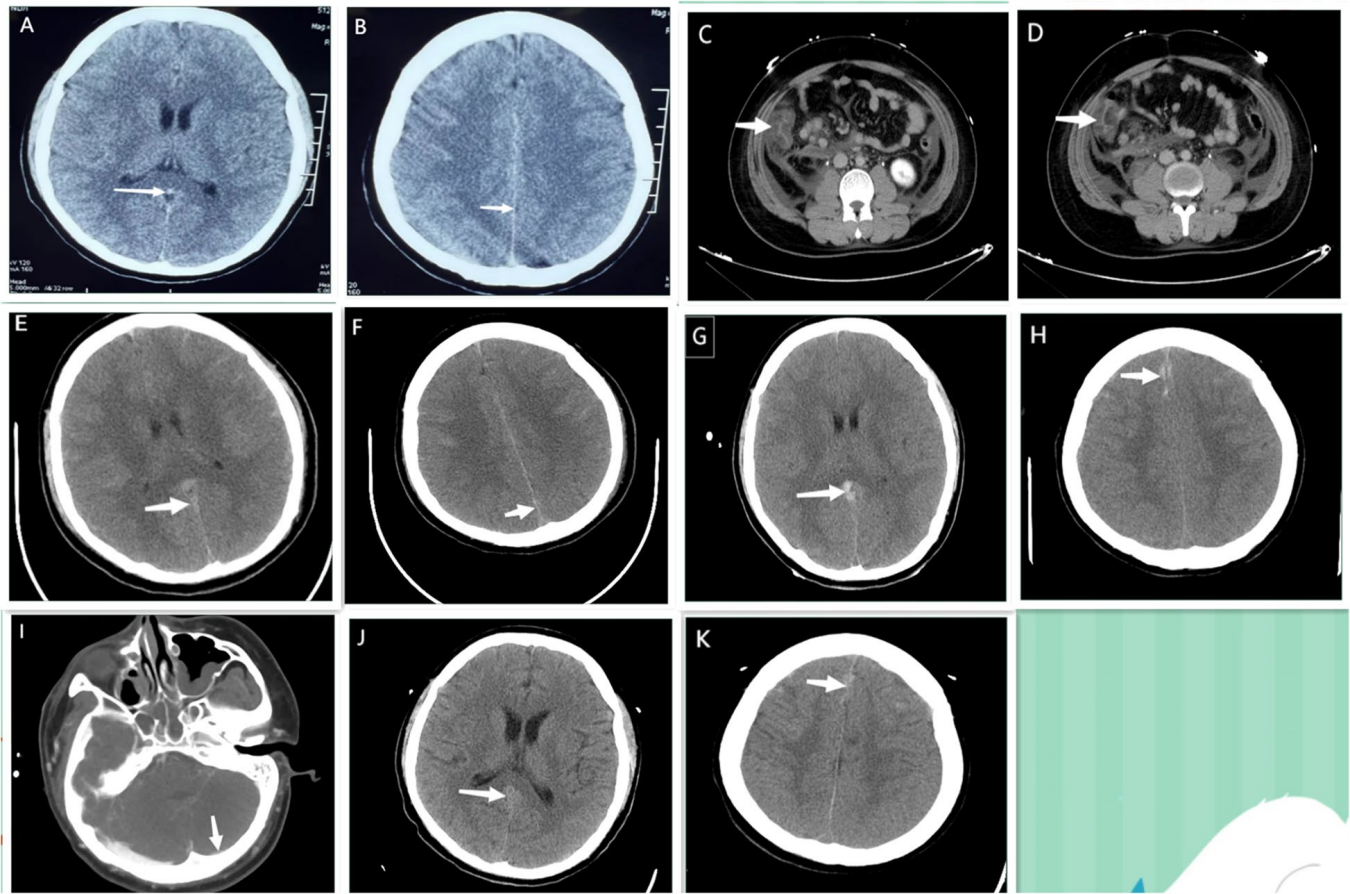
Radiographic findings of the patient. **A**-**B** showed cerebral edema and subarachnoid hemorrhage by cranial CT in local hospital; **C**-**D** showed local edema and thickening of the ascending colon wall with peritoneal thickening by abdominal CT plain scans in our hospital; E–F showed aggravated cerebral edema and subarachnoid hemorrhage by CT One day later; G-H showed significantly aggravated cerebral edema and multiple subarachnoid hemorrhages by CT After 12 days; I showed fine contrast in the left transverse sinus, sigmoid sinus and the opposite side of the internal jugular vein, and less uniform local density of transverse sinusAfter 12 days; J ~ K showed alleviated cerebral edema and subarachnoid hemorrhage after 2 weeks

A 20-year-old male college student visited the local hospital due to fever, disturbance of consciousness, and convulsion. He had abdominal distension, vomiting, and gradual abdominal pain without diarrhea after meal three days before. The patient had no meningeal irritation and no history of neurological disorder in his family. Physical examination on admission showed body temperature of 40.2 °C, blood pressure of 90/50 mmHg, and heart rate of 160 bpm. The patient developed a coma and abdominal distension of no reason. He became hypotensive and was commenced on norepinephrine treatment (Maximum dose reached 0.72ug/kg/min). Hematological examination revealed raised white blood cell (WBC) of 30.89 × 10^9^/L and D-dimer level of 13,484 ng/ml (normal value 0–243 ng/ml), slightly decreased blood platelet (PLT) of 75 × 10^9^/L. Coagulation tests suggested abnormal coagulation profile with prothrombin time (PT) of 14.5S (normal value 11.5–14.8S), activated partial thromboplastin time (APTT) of 35.8S (normal value 25.1–36.5S), thrombin time (TT) of 36.5 S (normal value 14-21S), fibrinogen (FIB) of 1.64 g/L (normal value 2.38–4.98 g/L), and fibrin degradation products (FDP) of 71.2ug/ml (normal value 0–2.01ug/ml). There were apparent liver and kidney dysfunction with alanine aminotransferase (ALT) of 131.6 U/L, aspartate aminotransferase (AST) of 111.3 U/L, total bilirubin (TBIL) of 33.8 μmol/L, creatinine of 125 μmol/L, and urea of 8.2 mmol/L. Laboratory investigations showed elevated level of C-reactive protein (CRP 221.85 mg/L) and procalcitonin (PCT 1.76 μg/L). But normal range of serum amylase and lipase, and the results of immunoglobulin lymphocyte subsets and connective tissue diseases are normal. CSF (cerebrospinal fluid) examination showed pale bloody CSF, red blood cells of 12,000 × 10^6^/L, white blood cell of 100 × 10^6^/L, with a pressure of 230cmH_2_O. Head CT performed at local hospital showed cerebral edema and subarachnoid hemorrhage (Fig. 1A and B). No abnormality was found in digital subtraction angiography (DSA) of skull. Purulent urine was drained from the indwelling urinary catheter. The patient was initially diagnosed with subarachnoid hemorrhage, sepsis, and septic shock. Therefore, meropenem (1.0 g, Q8h) and datomycin (0.5 g per day) were given for anti-infection, and anticoagulant therapy (enoxaparin 6000U, Q12h) was given, blood culture was also performed before antibiotics are used.

**Fig. 2:**
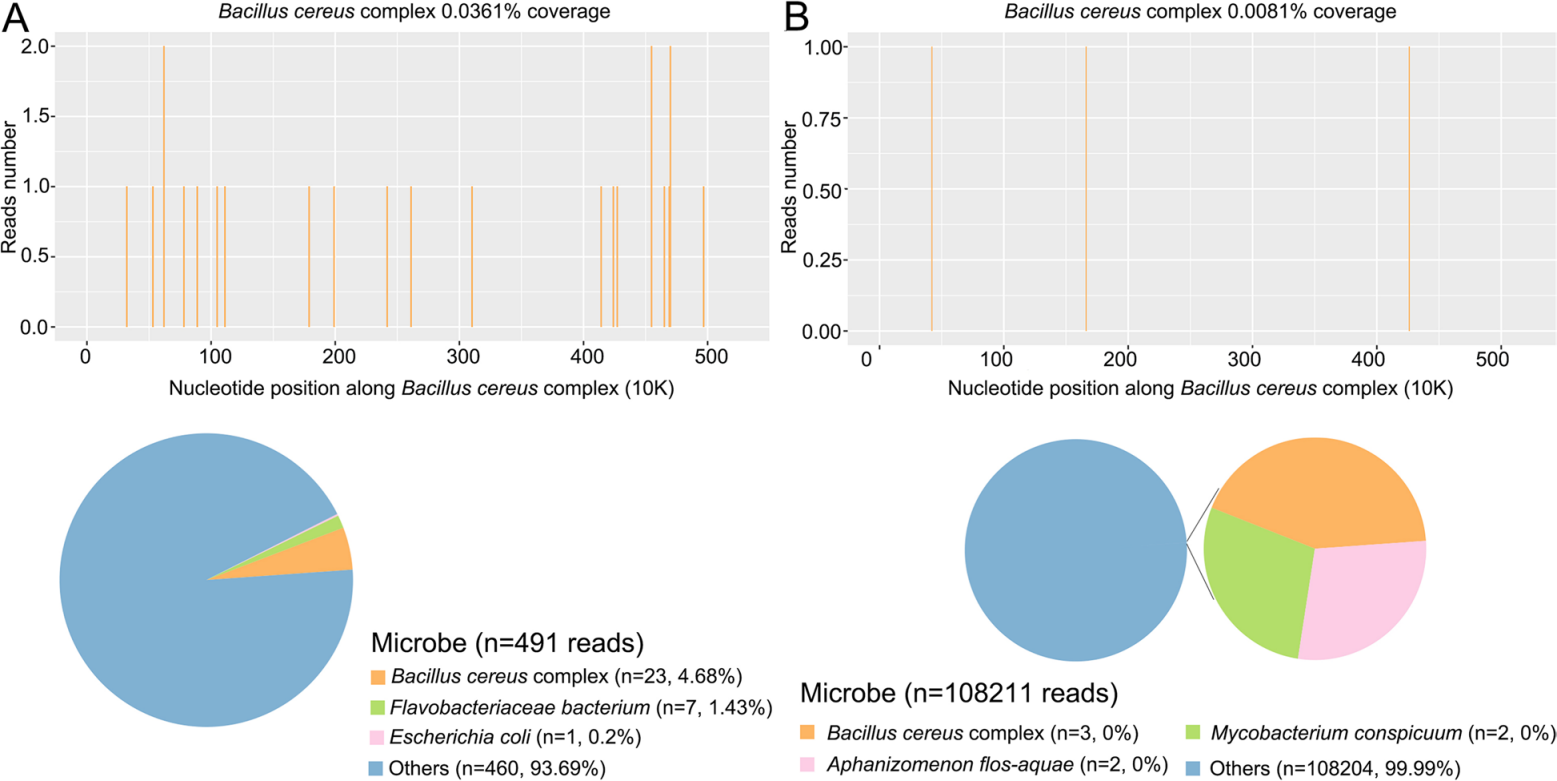
mNGS results of the patient. **A**. The DNA reads positions of *B. cereus* and the content of detected microbes by blood mNGS. The coverage of *B. cereus* detected by blood mNGS was 0.0361%. **B**. The DNA reads positions of *B. cereus* and the content of detected microbes by urine mNGS. The coverage of *B. cereus* detected by urine mNGS were 0.0081%

One day later, the patient was transferred to our hospital due to shock and multiple organ dysfunction. Abdominal CTA (computed tomography arteriography) and CTV (computed tomography venography) revealed no abnormalities, but local edema and thickening of the ascending colon wall with peritoneal thickening were found by abdominal CT plain scans (Fig. 1C and D). Head CT plain scans showed aggravated cerebral edema and subarachnoid hemorrhage (Fig. 1E and F). Lumbar puncture was not performed due to severe cerebral edema and high intracranial pressure. Four days later, both blood and urine were used for PACE seq mNGS (pathogen capture engine mutagenic next-generation sequencing), which detected 23 and 3 unique reads of *B. cereus*, respectively (Fig. 2A and B). Six days later, the blood culture results at the local hospital were reported as *Bacillus cereus*, and *the results of drug sensitivity report that Cefazolin, cefoperazone sulbactam, levofloxacin*, vancomycin *and linezolid are effective.*

Accordingly, the patient was diagnosed with *B. cereus* sepsis and sepsis-associated encephalopathy, complicated with multiple organ dysfunction, including gastroenteritis, acute kidney injury, urinary tract infection, and acute respiratory distress syndrome. Anticoagulation was promptly discontinued in view of the patient's increased subarachnoid hemorrhage. Sufficient meropenem (1.0 g, Q8h) combined with linezolid (0.6 g, Q12h) was administered for anti-infection. Adjuvant treatments, such as sedative and analgesic anti-sympathetic therapy, body temperature management, cerebral protection, assisted ventilation, hemoperfusion to remove inflammatory mediators, and volume management were also given. The patient's vital signs were gradually stabilized, and the laboratory test indicators showed improvement after a week. The patient had gastrointestinal bleeding during the treatment of patients, and was given symptomatic hemostatic treatment.

After 12 days, the patient developed increased heart rate (> 100 beats/min) and fever (39.2 °C). Reexamination of head CT showed significantly aggravated cerebral edema and multiple subarachnoid hemorrhages (Fig. 1G and H). Head CTA showed bilateral anterior, middle and posterior cerebral arteries with uneven thickness, multiple luminal narrowing, and dilatation changes, suggesting local cerebrovascular inflammation. CTV showed fine contrast in the left transverse sinus, sigmoid sinus and the opposite side of the internal jugular vein, and less uniform local density of transverse sinus (Fig. 1I). Venous sinus thrombosis was considered. Active adequate anticoagulation therapy was given, and because meropenem and linezolid had been anti-infective treatment for 2 weeks, considering the side effects of the drugs, the anti-infective treatment regimen was adjusted to cefoperazone/sulbactam (3.0 g, Q8h) combined with vancomycin (1.0 g, Q12h). Repeated head CT showed alleviated cerebral edema and subarachnoid hemorrhage after 2 weeks. (Fig. 1J and K).

**Fig. 3:**
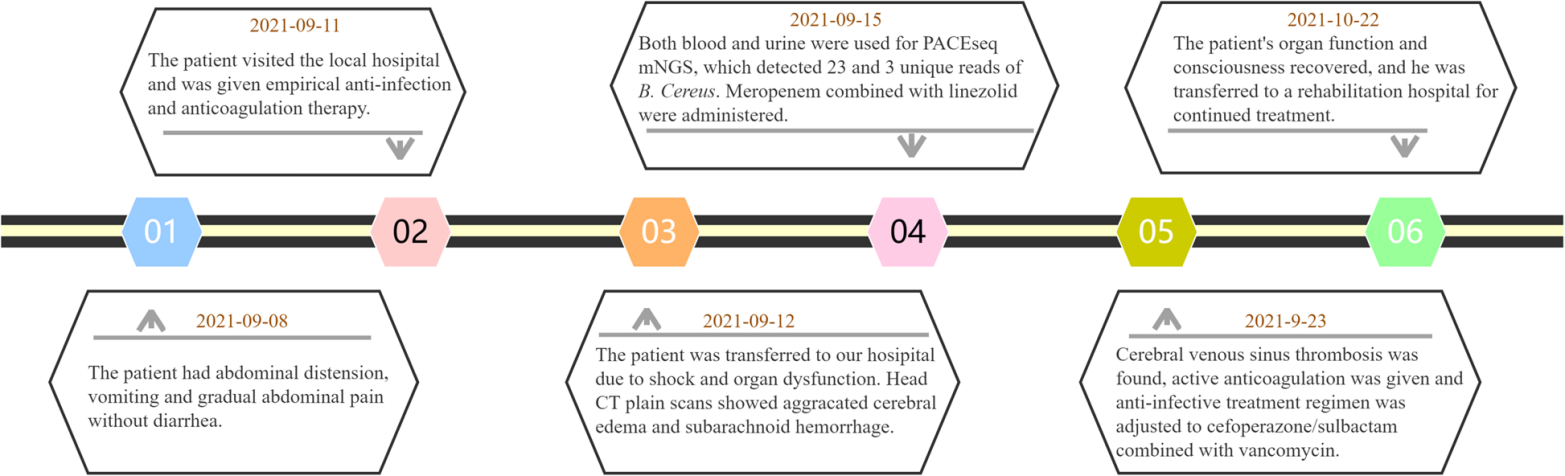
Timeline for the reported case

After forty days, the patient's organ function and consciousness recovered, but he was in a minimally conscious state, and his limbs could move spontaneously and still could not communicate. He was transferred to a rehabilitation hospital for continued treatment. During this period, lumbar puncture examination was performed intermittently, and no abnormality was found. After 2 months, the patient was able to communicate and take care of himself, but his cognitive function remained poor. After 4 months, his cognitive function gradually recovered. Six months later, he was able to return to school and continued his education. Clinical course timeline is described in Fig. 3.

## Discussion

*B. cereus* is widely distributed in various environments, such as contaminated meat, rice, and pasta of the living environment. Intravascular catheters, open wounds and contaminated sheets are the major medical environmental sources of *B. cereus* [5]. *B. cereus* is often responsible for self-limiting food poisoning, mainly characterized by diarrhea and vomiting, usually causing transient gastroenteritis in immunocompetent patients [6]. For immunosuppressed patients, *B. cereus* can cause not only gastrointestinal diseases, but also other infectious diseases such as fulminant bacteremia, central nervous system infections [7], endophthalmitis [8], osteomyelitis [9], urinary tract infections [10], skin infections [11], endocarditis [12], and pneumonia [13]. The rapid and fulminant progress of *B. cereus* caused illnesses seriously threatens the patients' life [14]. Currently, patients with hematological disorders, who received intravascular catheters, have become the high risk population for fulminant bloodstream and even blood-borne disseminated central nervous system infections caused by *B. cereus* [2, 15]. Severe illnesses with or without gastroenteritis caused by *B. cereus* in previously healthy individuals are uncommon. We reviewed the reported cases with *B. cereus* infections, who were previously healthy (Table 1), and found there were only five patients with bloodstream infections. In this case, the patient was previously healthy, who presented with discomfort after eating rice, severe *B. cereus* sepsis, accompanied by impaired consciousness and multiple organ dysfunction was considered.

**Table 1:** Summary of cases of *B. Cereus* reported in previously healthy individuals

No	Year	Sex	Age	Profession	Basic disease	Symptoms on presentation	Diagnosis	Pathogen detection method	Treatment (antibiotics)	Outcome	Reference (PMID)
1	1985	M	67	Engineer	cardiac failure	Retrosternal and right pleural pain	Pleuropulmonary infection	Pleural fluid cultures	GEN, CFZ, ERY	Death	3,936,280
2	1985	M	18	Student	No	Fever, cough, hemoptysis	Pneumonia	Sputum and blood cultures	CLDM, ERY, AMP	Survival	3,919,626
3	1991	M	21	Unknown	Bronchiectasia	Fever, cough, chills	Pneumonia	Sputum and BALF cultures	CPFX	Survival	1,902,995
4	1997	M	17	Student	No	Vomiting, lethargy, jaundice, abdominal pain	Fulminant hepatic failure, rhabdomyolysis, and acute renal failure	Intestinal contents and pan residue cultures	Untreated by antibiotics	Death	9,099,658
5	1997	M	46	Welder	No	Fever, cough, hemoptysis, vomiting	Bacteremia Pneumonia	Sputum and blood cultures	CPFX, CTX	Death	9,003,628
6	1997	M	41	Welder	No	Fever, cough, hemoptysis	Bacteremia Pneumonia	Sputum and blood cultures	AMP/SUL, ERY, CLDM, VAN	Death	9,003,628
7	2002	M	29	Unknown	No	Left leg erythema papules, ulceration	Skin soft-tissue infection	Tissue cultures	CPFX	Recovery	12140490 [11]
8	2003	M	76	Unknown	Abdominal operation	Fever and shock occurred 17 days after surgery	BSI	Blood culture and central venous tip cultures	PIP/TZP	Recovery	14,616,690
9	2008	F	69	Unknown	Diabetes, hypertension	Difficulty breathing, sweating, weight loss	Endocarditis	Blood cultures	CFZ	Recovery	18706120 [16]
10	2008	F	9	Student	No	Abdominal pain vomiting diarrhea, convulsions, coma, shock	Gastroenteritis, liver failure	Food cultures	MEM, CLDM	Recovery	18,664,929
11	2011	M	20	Student	No	Headache, nausea, vomiting, abdominal pain, diarrhea	Gastroenteritis, liver necrosis, colon necrosis	Faeces cultures	Untreated	Death	22,012,017
12	2010	M	11	Student	No	Abdominal cramps, vomiting, lethargy, convulsions	Gastroenteritis, liver failure, encephalopathy	Gastric juice, leftover race and faeces cultures	Untreated by antibiotics	Recovery	19796886 [17]
13	2011	M	74	Unknown	Valvular history, coronary stent history	Fever, dyspnea, night sweats, shock	BSI	Blood and bone marrow cultures	VAN, CPFX	Death	22173364 [9]
14	2016	M	59	Mechanic	No	Fatigue, nausea, indigestion, weight loss	Liver abscess	Puncture drainage fluid cultures	VAN, LEV	Recovery	28025629 [18]
15	2017	F	81	Unknown	Atrial fibrillation, Cardiac failure	Fever, cough, respiratory distress	Pneumonia, BSI	Blood sample cultures	IPM, LEV	Recovery	29457069 [19]
16	2019	F	32	Unknown	Abdominal trauma	Fever 48 h after surgery	Liver abscess, BSI	Drainage fluid and blood cultures	AMP/SUL, VAN	Recovery	31,791,818
17	2019	M	60	Unknown	Hypertension	Hemoptysis, respiratory distress, chest pain, right shoulder and right abdominal pain, numbness in the right hand	Necrotic hemorrhagic pneumonia	Sputum cultures	Untreated by antibiotics	Death	30813918 [13]
18	2019	M	91	Unknown	End-stage renal disease	High fever, shortness of breath, shock, ARDS	Aortic aneurysm	Blood cultures	VAN, CTRX,	Recovery	31,711,418
19	2020	M	58	School principal	Diabetes	Intermittent low fever, painless hematuria, oliguria, weight loss, focal epilepsy	Infective endocarditis, cerebral hemorrhage, splenic abscess	Identified by BD Phoenix	CPFX	Recovery	32655954 [12]
20	2020	F	40	Unknown	No	Vomiting, abdominal pain, syncope	Sepsis, multi-organ failure, shock, metabolic acidosis, rhabdomyolysis, coagulopathy,	Mass spectrometry (Bruker)	PIP/TZP, MNZ, AZI, VAN, MEM	Death	33462030 [20]
21	2021	M	25	Chef	No	Nausea, vomiting, diarrhea, abdominal pain	Gastroenteritis, liver failure	Leftover rice cultures	PIP/TZP	Recovery	35050989 [21]
22	2022	F	27	Unknown	No	Fever Abdominal pain vomiting diarrhea	Malaria, Gastroenteritis	Blood cultures	VAN/levofloxacin	Recovery	36,712,755
23	2022	No	80	Unknown	pituitary tumor	Abdominal pain vomiting diarrhea	meningoencephalitis	Blood cultures, broad-range 16 s rDNA PCR	VAN	Recovery	35,880,229
24	2022	F	33	Unknown	No	small vesicles, itching	cutaneous infection	broad-range 16 s rDNA PCR	CPFX	Recovery	35,448,975
25	2023	F	40	Unknown	type 1 bipolar disorder, major depressive disorder (MDD), migraines without aura, hypothyroidism,	severe headache	meningoencephalitis	Blood cultures, cerebrospinal fluid cultures	VAN	Survival	36,788,826

*AMP* Ampicillin, *AZI* Azithromycin, *AMP&SUL* Ampicillin/sulbactam, *CFZ* Cefazolin, *CTX* Cefotaxime, *CTRX* Ceftriaxone, *CLDM* Clindamycin, *CPFX* Ciprofloxacin, *ERY* Erythromycin, *GEN* Gentamicin, *IPM* Imipenem, *LEV* Levofloxacin, *MEM* Meropenem, *MNZ* Metronidazole, *PIP/TZP* Piperacillin/tazobactam, *VAN* Vancomycin

The pathogenicity and virulence of *B. cereus* are associated with toxins and enzymes production by this pathogen, including hemolytic, non-hemolytic enterotoxins, emetic toxins, and some enzymes that cause tissue necrosis [22]. The emetic toxin with a lipophilic ring structure belongs to mitochondrial toxins [23] and can resist heating and proteolysis, which can destroy mitochondrial function leading to impaired mitochondrial β oxidation process and then cause cell death and promote multiple organ dysfunction. A case of a chef with *B. cereus* infection caused by eating rice was recently reported, in which large amounts of *B. cereus* were detected in his leftover, and the non-ribosomal peptide synthase gene (*ces*) and the non-hemolytic enterotoxin gene (*nhe*) of the emetic toxin were detected. The patient's clinical presentation from initial vomiting and diarrhea to acute liver failure and renal tubular necrosis [21]. In addition, enzymes that promote tissue necrosis can also cause liver abscesses [15]. It is reported that the liver may become another target organ for *B. cereus* infection [18]. Fat droplet deposition is seen in liver biopsies of *B. cereus*-associated liver dysfunction, causing liver failure in severe cases [17, 21, 24]. In 1997, a 17-year-old boy was reported to have vomiting, diarrhea, and rapid development of liver failure and rhabdomyolysis after eating overnight spaghetti leftovers. Autopsy showed diffuse microbubble steatosis and intermediate zone necrosis in the liver [23]. Our patient presented with elevated liver enzymes and bilirubin after onset, and abdominal ultrasound and CT revealed diffuse abnormalities in the liver.

Emetic toxin of *B. cereus* can cross the blood–brain barrier to attack the central nervous system in immunocompetent people [20, 25], causing subarachnoid hemorrhage, parenchymal hemorrhage, brain abscess, cerebral edema, and disturbance of consciousness [15, 17]. Patients with hematological diseases combined with central nervous system involvement have a very poor prognosis for *B. cereus* infection [26]. For example, *B. cereus* has been reported to cause bacteremia in a patient with a history of neutropenic acute myeloid leukemia following chemotherapy, resulting in poor prognosis, including multiple parenchymal hemorrhages and subarachnoid hemorrhages in the brain, as well as diffuse intraparenchymal and subcortical liquefaction necrosis [27]. In 1995, a 26-year-old man with leukemia died after infection with *B. cereus*. Cellulose in the meningeal vessels and *B. cereus* in the meningeal vessels and intracranial necrotic indicated that *B. cereus* might trigger thrombosis [15]. This patient we reported was previously healthy and had no underlying diseases, such as hematological diseases. Early head CT showed cerebral edema and subarachnoid hemorrhage, but cranial DSA showed no vascular abnormalities. Anticoagulant therapy had been administered for suspected thrombotic disease due to the high level of D-dimer, which was later discontinued due to an expanding extent of subarachnoid hemorrhage. Later, head CTA and CTV examination revealed that there was indeed thrombus in the venous sinus. To our knowledge, this is the first case report of venous sinus thrombosis caused by community-acquired *B. cereus* infection in healthy adults with good outcomes.

Urinary tract infections caused by *B. cereus* have rarely been reported (only one case with invasive bladder cancer who underwent radical cystectomy with percutaneous left ureterostomy followed by irrigation of the ureter with saline) [10]. Our patient presented with pyuria drained from the urinary catheter after admission. Urine mNGS revealed *B. cereus,* consistent with blood mNGS detection, suggesting hematogenous systemic dissemination of bacteria from gastrointestinal infections. However, the patient's coagulation function did not deteriorate significantly, and the platelet count remained at 75 × 10^9^/L. The drained ascites was pale bloody, considering that *B. cereus* might cause systemic vascular injury, with multiple serous luminal hemorrhage, further observation was required.

*B. cereus* was highly susceptible to meropenem, linezolid and vancomycin [26, 27]. There are also reports of susceptibility of *B. cereus* to cefazolin [16], imipenem, levofloxacin [19], and ciprofloxacin [9]. The patient of this report was very young and had no underlying disease, with regular lifestyle. Systemic diseases rather than infection were considered due to the rapid progress and multiple organ dysfunction development. Fortunately, mNGS of blood and urine and blood culture detected the pathogen as *B. cereus.* Meropenem in combination with linezolid were subsequently given against *B. cereus*, which was later adjusted to cefoperazone/sulbactam in combination with vancomycin, resulting in good clinical outcome and normal final organ function as well as good recovery of consciousness. In addition, anticoagulation regimen against the definite venous sinus thrombosis of the patient also obtained good clinical effect. No similar treatment (anticoagulation regimen) has been reported at present, and this treatment may provide some reference for other cases.

This patient has severe sepsis with multiple organ dysfunction, it carries a risk for mortality, considerably exceeding that of a mere infection. Therefore, we actively give broad-spectrum antibiotics to fight infection and conduct bedside hemoperfusion treatment. There have been many literature reports on the clearance of inflammatory factors by hemoperfusion, and good results have been achieved[28, 29].

## Conclusions

In conclusion, we report a previously healthy, immunocompetent young male patient with community-acquired sepsis and sepsis-associated encephalopathy caused by *B. cereus*. Rapid and accurate pathogenic diagnosis by mNGS provides a significantly important role for the rapid diagnosis and timely adjustment of antibiotic treatment, saving the patient’s life. Appropriate anticoagulant therapy may help improve the conditions of patients with venous embolism caused by *B. cereus*. In addition, clinicians should raise vigilance for foodborne infections, fully recognize fulminant diseases caused by *B. cereus*.

## Data Availability

The authors are ready to provide the data in response to the need.

## Abbreviations

B.cereus: *Bacillus cereus*
MODS: Multiple organ dysfunction syndrome
mNGS: Metagenomic next-generation sequencing
CT: Computed tomography
CSF: Cerebrospinal fluid
CTA: Computed tomography arteriography
CTV: Computed tomography venography
DNA: Deoxyribo nucleic acid
PACEseq mNGS: Pathogen capture engine metagenomic next-generation sequencing
DSA: Digital subtraction angiography
